# Repaglinide-loaded nanostructured lipid carriers with different particle sizes for improving oral absorption: preparation, characterization, pharmacokinetics, and *in situ* intestinal perfusion

**DOI:** 10.1080/10717544.2019.1689313

**Published:** 2019-11-15

**Authors:** Lei Wu, Lin Zhao, Xitong Su, Peng Zhang, Guixia Ling

**Affiliations:** Wuya College of Innovation, Shenyang Pharmaceutical University, Shenyang, China

**Keywords:** Different sizes, NLCs, pharmacokinetics, SPIP, repaglinide

## Abstract

Repaglinide-loaded nanostructured lipid carriers (REP-NLCs) with different particle sizes were successfully designed and prepared to investigate the permeation and absorption ability by *in situ* single-pass intestinal perfusion (SPIP) study and pharmacokinetics. Both of the formulations prepared by solvent diffusion method exhibited a spherical shape under transmission electron microscopy (TEM) and similar zeta potential value of –11 mV. The particles size, encapsulation efficiency (EE), drug loading (DL) of REP-NLCs-Small and REP-NLCs-Large size preparations were about 79 nm and 325 nm, 96.83% and 98.60%, 4.41% and 3.05%, respectively. Besides, both REP-NLCs showed good colloidal stability and had no burst release phenomenon compared with REP-Sol. SPIP demonstrated the improved membrane permeability for NLCs compared with REP-Sol, especially NLCs-Small size preparation. The bioavailability increased sequentially in REP-Sol, REP-NLCs-Large, and REP-NLCs-Small, and the difference between each other was statistical significant. Our investigations demonstrate that NLCs with small particles size of 50–100 nm, such as 79 nm, are able to enhance absorption performance of a poorly soluble repaglinide compared with large particles size, such as 325 nm, by significantly improving the absorption in jejunum, and colon of rats and thus well improving oral bioavailability.

## 1. Introduction

NPs of oral administration have to overcome mucus diffusion barrier and epithelium absorption barrier except for ensuring NPs ability in gastrointestinal tract. The former requires relatively electro-neutral, hydrophilic surface of particles as well as size less than 200 nm (Lai et al., 2009; Shan et al., 2015) and will rapidly remove particles with cationic as well as hydrophobic nature (Lai et al., 2009; Dhuria et al., 2010; Shan et al., 2015). Influence factors of cellular uptake include drug property, nanomaterial, cellular type and density, particle size, surface charge, surface-modified ligands, and surface property (Bareford & Swaan, 2007; Harush-Frenkel et al., 2008; Doherty & McMahon, 2009; Hillaireau & Couvreur, 2009; Sahay et al., 2010; Shinde et al., 2011; Zaki et al., 2011; Kou et al., 2013; Xie et al., 2014). Generally, researchers consider that cationic and hydrophobic surface promote the interaction between particles and cell membrane. But, it have to be considered that NPs with negative charge have no obvious cytotoxicity while the positive charge will show low toxicity due to the biocompatibility (Huehn et al., 2013; Song & Chen, 2015; Sun et al., 2017). Besides, Lerch et al., (2013) researching positively charged polystyrene particles found that when above 200 nm the effect of surface area is not important significantly. Taking particle size, surface charge and NPs with hydrophilic surface into account, we try to prepare formulations of NLCs with similar negative charge, hydrophilic surface, and different size with the boundary at 200 nm.

Many researchers think the particle size is smaller, uptake is easier (Davda & Labhasetwar, 2002; Pantarotto et al., 2004; Soe et al., 2019; Yu et al., 2019), but, the excessively small size may likely be excreted and thus the total amount of particles is possible fewer. For example, US-SWNT with an average length of 30 nm was rapidly excreted by non-polarized human cervical epithelioid carcinoma cells HeLa and hepatoma cells (Donkor & Tang, 2014). Besides, we should consider that NPs below 10 nm are likely to pass through glomerular capillary to be eliminated by kidney (Yoo et al., 2011). Therefore, what is the optimal particle size for transporting across normal cells? Some research results may give us inspiration. Small particles (less than 100 nm) as well as uniform size distribution may have higher uptake efficiency (Desai et al., 1996). For NLC about 100 nm size is suitable for efficient uptake (Zhang et al., 2014). Particles size with <100 nm and a hydrophilic surface increase the possibility to escape recognition of opsonin (Feng, 2004). The intracellular localization of polylactic-co-glycolic acid (PLGA) NPs of 100 nm size range was in organelles (such as nucleus) while in lysosomes at 266.8 nm (Nkabinde et al., 2014). To enhance enhanced permeability and retention (EPR) effect, we should ensure size of <200 nm (Maruyama, 2002; Yoo et al., 2011) while the intercellular space of tumor tissue with EPR is bigger than normal cells. Therefore, we try to keep the influence factors of cellular uptake unchanged except for particle size and prepare NLCs with size 50–100 nm and size >250 nm.

Repaglinide, belonging to class II Biopharmaceutical Classification System (BCS II), is an oral medicine used to treat type 2 diabetes with very low aqueous solubility, poor oral bioavailability, and short terminal elimination half-life (Culy & Jarvis, 2001). Considering this drug needing to be taken for a long period and patient compliance, we try to prepare orally administrated repaglinide-loaded NLC to decrease dosage and side effect related to the drug.

In this study, different particles size repaglinide-loaded NLCs as a model of oral administration were prepared to investigate the effect of particles size on bioavailability and intestinal absorption. Besides, we prepare repaglinide-loaded NLCs using glyceryl monostearate and medium-chain triglyceride (MCT) as carriers for the first time and intestinal absorption investigation of repaglinide by *in situ* single-pass intestinal perfusion (SPIP) technique was carried out utilizing gravimetric method to correct drug concentration for the first time. The prepared NLCs were characterized by particle size, polydispersity index, zeta potential, morphology, encapsulation efficiency (EE), and drug loading (DL), and besides, the colloidal stability of prepared NLCs in differently simulated fluids was also investigated. Finally, *in situ* SPIP was used to study the intestinal permeability of prepared NLCs and pharmacokinetics was carried out to evaluate their oral absorption performances *in vivo* in rats. This study demonstrates the possibility to overcome mucus and epithelium barriers meantime for orally administered NPs.

## 2. Materials and methods

### 2.1. Materials

Repaglinide raw material was obtained from Tianjin Jinkang Pharmaceutical Ltd. (Tianjin, China). The commercial tablet of repaglinide was purchased from Novo Nordisk Co. (Bagsvaerd, Denmark). MCT was obtained from Lipoid GMBH (Ludwigshafen, Germany). Glycerin monostearate and Tween 80 were purchased from Tianjin Bodi Chemical Company (Tianjin, China). Poloxamer 188 (F68) was provided by BASF SE (Shanghai, China). Sodium dodecyl sulfate (SDS) was obtained from Anhui Shanhe Pharmaceutical Excipients Co., Ltd. (Huainan, China). Pepsin was purchased from Shanghai Macklin Biochemical Co., Ltd. (Shanghai, China). Trypsin was obtained from Dalian Meilun Biological Technology Co., Ltd. (Dalian, China). All other reagents used in this study were high-performance liquid chromatography (HPLC) grade.

### 2.2. Preparation of NLCs with different particles size

Repaglinide-loaded nanostructured lipid carriers (REP-NLCs) were prepared by solvent diffusion method. Optimal amounts of MCT, glycerin monostearate, and repaglinide were dissolved in 3 ml (5 ml in formulation with larger size) ethanol at 75 °C under stirring. In addition, 10 ml aqueous solution containing 0.5% F68 (w/v) (1% Tween 80 in formulation with larger size) was heated to the same temperature with the same stirring rate. After some time, the organic phase was dispersed quickly in aqueous phase. The mixed liquid was stirred for 20 min (or 5 min in formulation with larger size) to get emulsion or pre-emulsion which was sonicated at 237.5 W (or 285 W with larger size) for 8 min (or 6 min in formulation with larger size) with 2 s/4s (time of supersonic/time of intervals). Then, the solution was rapidly transferred into a brine bath with –20 °C to solidify the lipid droplets (stirring 25 min before this step in formulation with larger size).

### 2.3. Characterization of NLCs *in vitro*

#### 2.3.1. Particle size, zeta potential, and polydispersity index

The particle size, zeta potential, and polydispersity index (PDI) were measured by a Zetasizer (Nano ZS, Malvern Co., Malvern, UK), which was based on a technique of dynamic light scattering.

#### 2.3.2. Encapsulation efficiency and drug loading

EE and DL of NLCs were evaluated by determining the total amount of REP in NLC suspensions and encapsulated REP in NLCs. 0.8 μm membrane was used to remove free REP from NLC suspensions. The content of total REP in NLC suspensions and the encapsulated REP in NLCs was determined after ethanol disruption and deionized water with equal volume dilution by HPLC. The EE and DL of the NLCs were calculated using Equations (1) and (2), respectively.

(1) $$\text{EE}\left(\%\right)=W_{1}/W_{2}\times 100\%$$

(2) $$\text{DL}\left(\%\right)=W_{1}/\left(W_{1}+W_{3}\right)\times 100\%$$

where *W*_1_, *W*_2_, and *W*_3_ represent the number of REP encapsulated in the NLCs, the total amount of REP in NLCs suspensions, and the weighing of excipients, respectively.

#### 2.3.3. Colloidal stability and morphological observation

To investigate the colloidal stability of NLCs in different media, 200 μl of NLCs was added to 2 ml of different media including pH 1.2 hydrochloric acid, pH 6.8 phosphate buffer, pH 7.4 phosphate buffer, fetal bovine serum (FBS), simulated gastric fluid (SGF), and simulated intestinal fluid (SIF). The mixtures were incubated in a water bath at 37 °C with constant shaking. At 0, 2, 4, 6, 8, 12, 24 h, and the average particle size was determined by Zetasizer (Nano ZS, Malvern Co., Malvern, UK). In addition, the stability of REP-NLCs in seven days following storage at 4 °C was evaluated by measuring the particle size by Zetasizer (Nano ZS, Malvern Co., Malvern, UK). The morphology of REP-NLCs was observed by JEM-2100 transmission electron microscope (TEM, JEOL, Tokyo, Japan).

#### 2.3.4. *In vitro* drug release

*In vitro* release of REP-NLCs and REP-Sol was investigated by dialysis bag diffusion technique. pH 7.4 phosphate buffer was utilized as release media, with 0.01% SDS (w/v) to achieve the sink condition. One milliliter REP-NLCs of 0.6 mg REP or REP-Sol with equal amount of REP dissolved by ethanol was dialyzed against 50 ml media using a dialysis membrane (MW cutoff 12–14 kDa) in water bath at 37 °C with constant shaking. At 0.25, 0.5, 0.75, 1, 2, 4, 6, 8, 12, and 24 h, dialyzed solution of 1 ml was withdrawn and the fresh media of same volume was added to maintain the volume. The concentration of REP was determined by HPLC.

### 2.4. Animals

Sprague-Dawley (SD) rats were obtained from the Laboratory Animal Center of Shenyang Pharmaceutical University (Shenyang, China). All the animal experiments in this study were carried out according to the guidelines for the Care and Use of Laboratory Animals approved by the Institutional Animal Ethical Care Committee (IAEC) in Shenyang Pharmaceutical University. Before experiments, the rats were fasted for 12 h with free access to water.

### 2.5. *In vivo* pharmacokinetics

#### 2.5.1. Pharmacokinetic study of REP in rats

Twelve rats weighing 210–240 g were randomly divided into three groups. A single dose of 1.32 mg/kg commercial tablets of REP and REP-NLCs (equivalent to 1.32 mg/kg REP) was orally administered by an oral gavage by a stainless steel with a blunt end. At 0.083, 0.167, 0.250, 0.333, 0.500, 0.750, 1, 2, 4, 6, 8, and 12 h, blood of about 300 μl was collected from orbit and centrifuged at 13,000 rpm for 10 min to obtained plasma samples. The samples were stored at –20 °C until analysis.

#### 2.5.2. Preparation of plasma sample

One hundred microliters internal standard (IS) (gliclazide, 60 ng/ml in methanol) and 100 µl methanol were added to 100 µl rat plasma sample. Then, 100 µl of 0.05 M H_3_PO_4_ solution and 100 µl deionized water were added. The mixture was vortexed for 1 min and extracted with 3 ml of n-hexane–ether absolute (1:4, v/v) by vortexing for 3 min. After centrifugation at 3500 rpm for 10 min, the organic phase was transferred to another tube and evaporated to dryness at 37 °C under a stream of nitrogen. The residue was reconstituted using 100 µl of the initial mobile phase and then vortexed for 10 min. A 5 µl supernate after centrifuging at 13,000 rpm for 10 min was injected into the UPLC–MS–MS system.

#### 2.5.3. UPLC–MS/MS method

The concentration of REP in rat plasma was determined using a validated UPLC–MS/MS method after liquid–liquid extraction by ether absolute and n-hexane (4:1, v/v) with gliclazide as IS. The chromatographic separations were carried out on an ACQUITY UPLC^TM^ system (Waters Co., Ltd., Milford, MA) with a Phenomenex Kinetex C_18_ (50 mm × 2.1 mm, 2.6 μm). A binary gradient elution was performed at a flow rate of 0.2 ml/min. The solvent A was methanol and solvent B was aqueous solution with 0.05% formic acid and 0.1% acetic acid. The mobile phase started at 60% of solvent A and kept 0.6 min and increased to 85% solvent A using 0.02 min, holding this state until 2.2 min. It was then decreased to 60% solvent A at 2.22 min and allowed to equilibrate at 60% solvent A to 3.4 min. The total run time was 3.4 min. The injection volume was 5 μl and the column temperature was 35 °C. Mass spectrometric detection was operated with a triple quadrupole detector with electrospray ionization in positive ion mode (ESI^+^) and quantitative analysis with multiple reaction monitoring (MRM). The ion pairs used for monitoring were 453.4 → 230.3 for REG and 324.3 → 127.2 for gliclazide (IS). The mass spectrometric parameters were optimized and set as follows: cone voltage: 40 V (REG) and 35 V (IS); collision voltage: 25 V (REG) and 19 V (IS); capillary voltage: 3.5 kV; source temperature: 120 °C; desolvation temperature: 350 °C; desolvation gas flow: 550 L/h; cone gas flow: 50 L/h.

#### 2.5.4. Data analysis

The main pharmacokinetic parameters were calculated by DAS 2.1.1 software. The plasma concentration–time curves were obtained by origin 8.0 software.

### 2.6. *In situ* single-pass intestinal perfusion study

The previous SPIP studies about repaglinide are relatively infrequent except for Yin et al. investigating recirculating perfusion by phenol red to correct (Yin et al., 2012). In this study, intestinal perfusion determining intestinal absorption performance of repaglinide was carried out by SPIP study utilizing gravimetric method to correct drug concentration for the first time according to previous study and pretest experiments (Sun et al., 2016; Dezani et al., 2017). REP-NLCs and REP solution were diluted to 10 μg/ml, calculated by considering drug dosage in pharmacokinetic study and volume of intestinal perfusion, by pH 6.8 Krebs-Ringer’s (K-R). Rats with 270–300 g were anesthetized by intraperitoneal injection with urethane (1 g/kg) and then were restrained in a supine position. Upon the loss of pain reflex response, 10 cm segment of duodenum or jejunum or ileum or 6 cm colon was exposed by the midline abdomen incision of 1–3 cm and then was cannulated as well as ligated at both ends. After gentle rinsing with 37 °C saline for about 10 min, the segment was equilibrated with perfusates for 40 min at a flow rate of 0.2 ml/min. Then the perfusion experiment continued 105 min to collect seven perfusate samples. Moisten the exposed abdominal cavity using warm saline solution to prevent issue dry. The decreased weight of donor flask and increased weight of receptor vial were accurately weighed and calculated every 15 min. The concentration of repaglinide was determined by HPLC after diluting samples by ethanol to disrupt NLCs. Finally, the rats were euthanized, and the diameter and length of each infused segment were measured. The absorption rate constant (*K*_a_) and effective permeability (*P*_eff_) of repaglinide were calculated according to the following equations:

(3) $$K_{a}=\left(1-\frac{C_{\text{out}}}{C_{\text{in}}}\cdot \frac{V_{\text{out}}}{V_{\text{in}}}\right)\cdot \frac{Q}{\pi r^{2}l}$$

(4) $$P_{\text{eff}}=\frac{-Q\cdot \;\text{ln}\;\left(\frac{C_{\text{out}}}{C_{\text{in}}}\cdot \frac{V_{\text{out}}}{V_{\text{in}}}\right)}{2\pi rl}$$

*C*_out_ and *C*_in_ are the repaglinide concentration in the outlet and inlet of perfusates, respectively; *V*_out_ and *V*_in_ are the volume of perfusates in the receptor vial and donor flask, respectively and the density of the perfusates was 1 kg/l by determining; *Q* is the flow rate with constant 0.2 ml/min; *l* is the length of intestinal segment (cm) and *r* is the radius of intestinal segment (cm).

### 2.7. Statistical analysis

All quantitative data were shown as means ± standard deviation (SD). One-way ANOVA was used to evaluate differences among multiple groups in the experiments by variance analysis in the SPSS software (version 19, IBM SPSS Statistics, Armonk, NY). *p < .05 was thought to be statistically significant difference, and **p < .01 was thought to be very significant difference.

## 3. Results and discussion

### 3.1. Preparation of NLCs with different particle sizes

We successfully prepared NLCs with different particles size by solvent diffusion method. Ethanol is nontoxic and is easily soluble and diffused in water with minimal interfacial tension. In our investigations, type of stabilizer, volume ratio of organic phase and aqueous phase, stirring time before sonication and sonication time could affect the particle size. Besides, the heated temperature with properly upper than melting point of solid lipid and low cooling temperature contributed to get small and stable particles. We had to note that the length of ultrasonic probe in solution and diameter of ultrasonic tube were important for particle size and homogeneity of particles. The NLCs with oleic acid as liquid lipid were instable and would precipitate after one day, which may be due to the incompatibility between oleic acid and repaglinide. We could not get stable colloidal solution no matter that the liquid lipid was oleic acid or MCT, and the stabilizers was Tween 80, F68, Span 80, lecithin, or their mixture with proper HLB value and different ratios.

NLCs consist of nonirritating, biodegradable, and biocompatible solid as well as liquid lipids (Gaba et al., 2015). At first, we tried to prepared different particles size NLCs with the same stabilizer, but lecithin or Span 80 or mixed with F68 was instability, and besides, the stability of small particle size with Tween 80 was worse than F68 and could not prepare NLCs of more than 300 nm size using F68 by changing varieties of factors. The influence of NPs with different stabilizers on permeation absorption of NPs is not certain due to the few investigations and seemingly conflicting results. For example, Liu et al. prepared PTX/F127 (1/5) nanocrystals with 105.8 nm and PTX/TPGS (1/5) nanocrystals with 77.84 nm and found that they had no significant differences in biodistribution in major organs and in tumor-bearing mice (Liu et al., 2016), and besides, Elnaggar et al. fabricated daidzein complex-loaded self-emulsifying phospholipid preconcentrates (SEPPs) with or without surfactant Tween-80/Transcutol HP and both of them had no significant differences in oral bioavailability, revealing surfactant Tween-80/Transcutol HP having no effect on the absorption of daidzein complex-loaded SEPPs (Elnaggar et al., 2017). However, Mu et al. investigated the pharmacokinetic performances of spironolactone nanocrystals with similar particles size and different stabilizers F127 or F68 or HPMC-E5 and found that stabilizers can affect *in vivo* performances (Mu et al., 2016). It was interesting that the surface characterization, such as charge, can be different for different particles size even though prepared SLNs with same stabilizer F68 (Sanjula et al., 2009). These seemingly conflicting results may be attributed to varieties of factors of prepared NPs, such as particle size, zeta potential, surface structure properties, stability in physiological media, *in vitro* release characterizations, appearance, and so on. Therefore, considering that both F68 and Tween 80 used in this investigation were nonionic surfactants, we tried our best to make different particles size NLCs with similar zeta potential, shape, stability, and *in vitro* release.

### 3.2. Characterization of REP-NLCs

#### 3.2.1. Particle size, zeta potential, PDI, EE, and DL

The particle size, zeta potential, EE, and DL of blank NLC with large size, blank NLC with small size, REP-loaded NLC with large size and REP-loaded NLC with small size are listed in Table 1. The REP-NLCs had similar zeta potential and particle size with corresponding blank NLCs, demonstrating that the participation of REP had no effect on the zeta potential and particle size. Besides, the REP-NLCs-Small and REP-NLCs-Large had similar zeta potential value, showing similar surface charge between these two kinds of preparations.

**Table 1.** Characterization of REP-NLCs-Small, REP-NLCs-Large, and corresponding blank NLCs (mean ± SD, *n* *=* 3).

Nanoparticles	Particle size (nm)	PDI	Zeta potential (mV)	Encapsulation efficiency (%)	Drug loading (%)
Blank NLCs-Large	351.0 ± 16.55	0.223 ± 0.171	–3.49 ± 1.50	–	–
Blank NLCs-Small	76.90 ± 2.613	0.260 ± 0.007	–10.2 ± 2.08	–	–
REP-NLCs-Large	325.3 ± 6.090	0.152 ± 0.083	–10.9 ± 2.35	98.60 ± 0.26	3.05 ± 0.01
REP-NLCs-Small	79.83 ± 3.895	0.275 ± 0.004	–12 ± 2.89	96.83 ± 0.55	4.41 ± 0.003

#### 3.2.2. Colloidal stability and morphology observation

**Figure 1.**
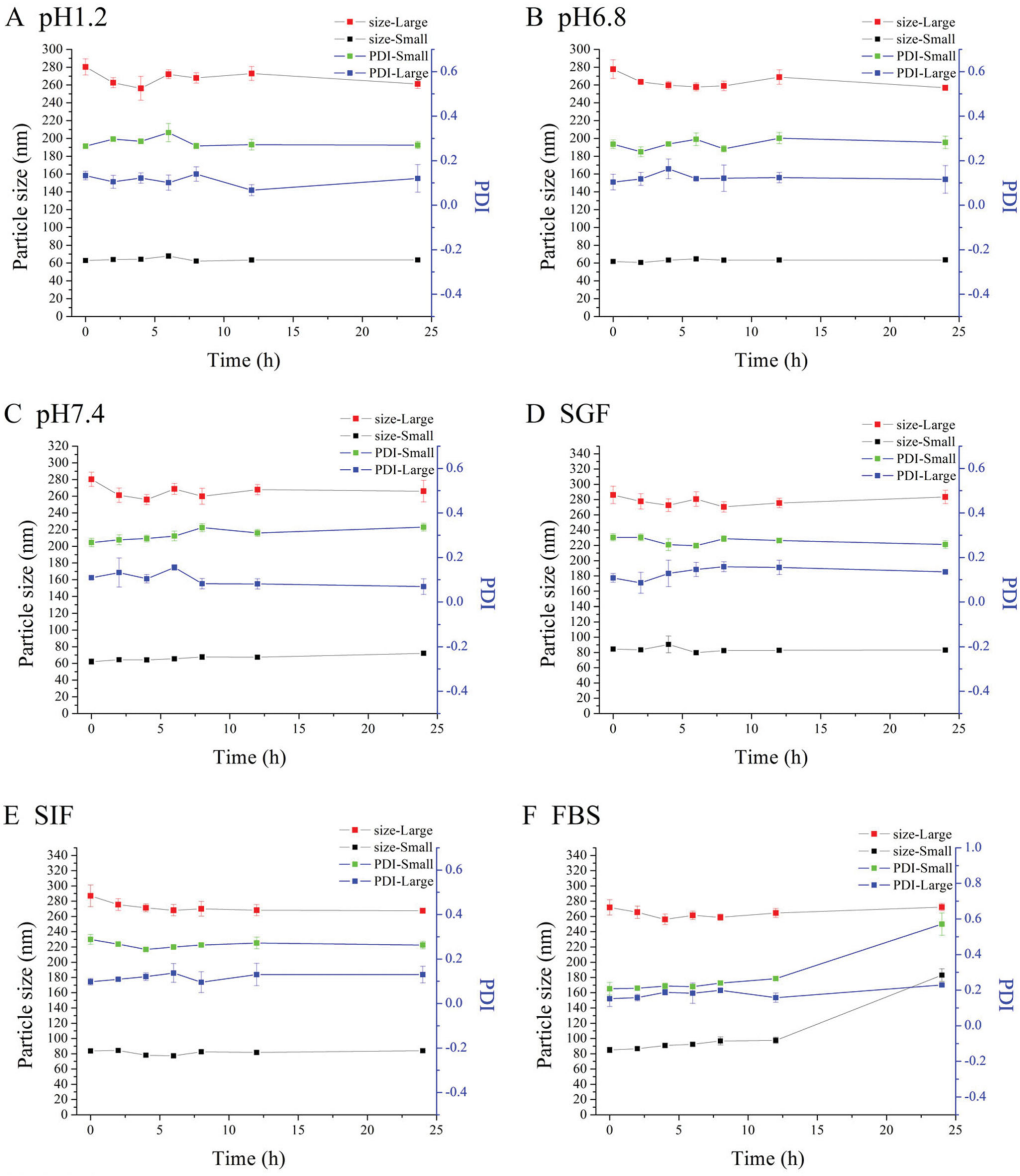

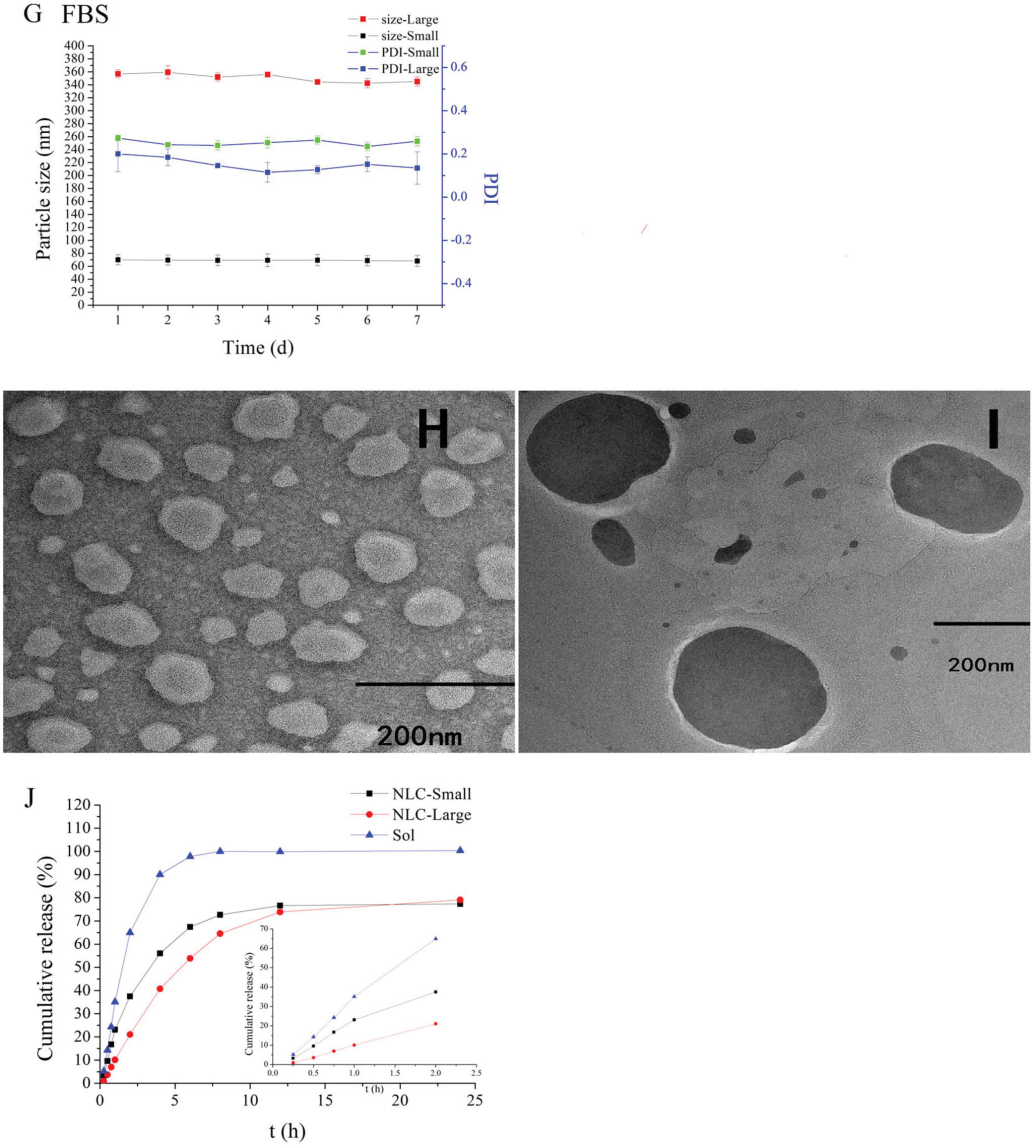
Characterization of NLCs. The particle sizes and PDI changes of REP-NLCs-Small and REP-NLCs-Large in pH 1.2 hydrochloric acid solution (A), pH 6.8 phosphate buffer (B), pH 7.4 phosphate buffer (C), SGF (D), SIF (E), FBS for 24 h (F), and at 4 °C for 7 days (G), respectively (mean ± SD, *n* = 3). The TEM images of REP-NLCs-Small (H) and REP-NLCs-Large (I), respectively. The *in vitro* release profiles of REP-NLC-Small, REP-NLC-Large and REP-Sol (J, mean ± SD, *n* = 3).

The particle size and PDI had few changes in different media except for REP-loaded NLC with small size at 24 h in FBS (Figure 1(A–G)). We could consider that all NLCs were stable during all experiments according to the results because the half-life period of repaglinide is short and the concentration approached to zero at 12 h *in vivo* in rats. TEM images showed that both the small size NLC and large size NLC were sphere-like shape and the particles size were similar and slight small to the results of determining by Zetasizer (Nano ZS, Malvern Co., Malvern, UK) due to the shrinkage of hydration layer probably, and besides, the distribution of particles size was homogeneous (Figure 1(H–I)).

#### 3.2.3. *In vitro* drug release

The cumulative release profiles of REP-Sol, REP-NLCs-Small, and REP-NLCs-Large are shown in Figure 1(J). About 90% REP was released for REP-Sol at 4 h, while about 70% REP was released for both REP-NLCs-Small and REP-NLCs-Large at 24 h. The release rate of REP-NLCs-Small was faster than REP-NLCs-Large at first, which may be because of small size and larger contact surface area in REP-NLCs-Small size (Ebrahimi et al., 2015). It was clear that both REP-loaded NLCs had no burst release phenomenon, indicating encapsulation of REP in the NLCs, rather than on the surface of NLCs.

Results showed that repaglinide was encapsulated in NLCs and two kinds of REP-NLCs were stable in our studied conditions and had no burst release phenomenon compared with REP-Sol, which laid the foundation for the following SPIP study and pharmacokinetics. Besides, we have to note that zeta potential values were small and negative close to electro-neutral and similar to the NPs permeating across mucus well of Shan et al. (2015), which were beneficial for mucus penetration and cellular internalization compared with higher negative charge.

Therefore, we can think that the difference of two kinds of NLCs is mainly particle size and they both may be easy to pass mucus considering low negative-charge surface as well as hydrophilicity.

### 3.3. Pharmacokinetic study

**Figure 2.**
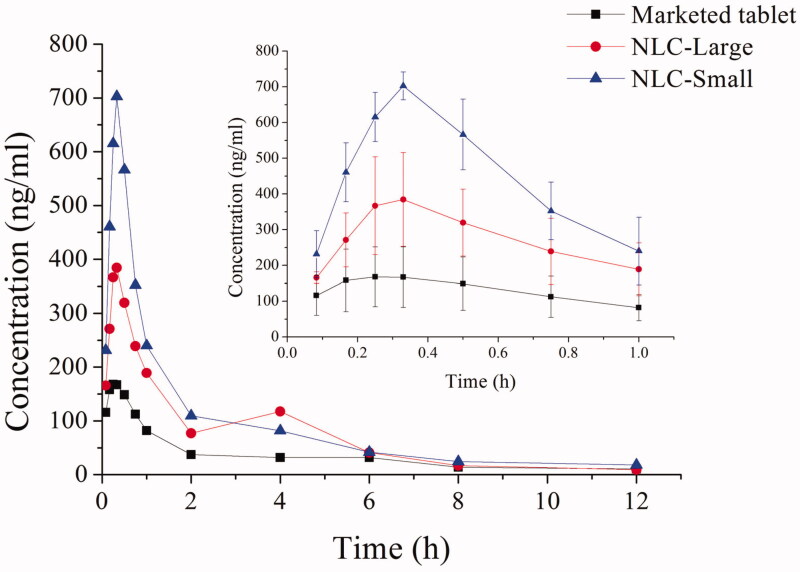
The mean plasma concentration–time curves of REP-NLCs-Small, REP-NLCs-Large, and REP-marketed tablets by orally administrated 1.28 mg/kg of repaglinide in rats (mean ± SD, *n* = 4).

The pharmacokinetic study of marketed tablet of repaglinide and REP-NLCs after single-dose oral administration was investigated in SD rats. The plasma concentration of repaglinide from high to low was REP-NLC-Small size, REP-NLC-Large, marketed repaglinide observing from Figure 2, demonstrating good absorption ability *in vivo* for NLCs, especially for REP-NLC-Small size. The pharmacokinetic parameters are listed in Table 2. Compared with marketed repaglinide, the AUC_(0→_*_t_*_)_ of REP-NLC-Small size and REP-NLC-Large size were increased to 3.02-fold (*p* < .01) and 1.93-fold (*p* < .01). The *C*_max_ of REP-NLC-Small size and REP-NLC-Large size was also significantly increased than marketed repaglinide tablets (*p* < .01 for both NLCs). Besides, compared with REP-NLC-Large size, REP-NLC-Small size showed significantly better bioavailability and *C*_max_ improvement (*p* < .01). The three groups had no significant difference in *T*_max_ and *T*_1/2_ (*p*> .05).

**Table 2.** Pharmacokinetic parameters in each group.

Parameters	Marketed tablets	NLC-Large	NLC-Small
*AUC_0–12_* (μg/l·h)	355.717 ± 55.992	685.899 ± 92.459**	1073.958 ± 175.391**
*C*_max_ (μg/l)	195.1 ± 84.217	523.125 ± 94.500**	702.8 ± 38.988**
*T_1/2_* (h)	6.148 ± 3.612	5.336 ± 3.927	3.401 ± 1.26
*T*_max_ (h)	0.27 ± 0.158	0.27 ± 0.04	0.33 ± 0.000

^**^ *p*< .01 for REP-NLCs-Large versus REP-marketed tablets and REP-NLCs-Small versus REP-NLCs-Large and (mean ± SD, *n=* 4).

### 3.4. *In situ* single-pass intestinal perfusion study

The *in situ* intestinal perfusion is thought the most similar to *in vivo* conditions, such as innervation, intact blood supply, active enzymes and transporters (Dezani et al., 2017) and is reliable for prediction of absorption performance, permeability mechanisms, and BCS classification (Lozoya-Agullo et al., 2015).

**Figure 3.**
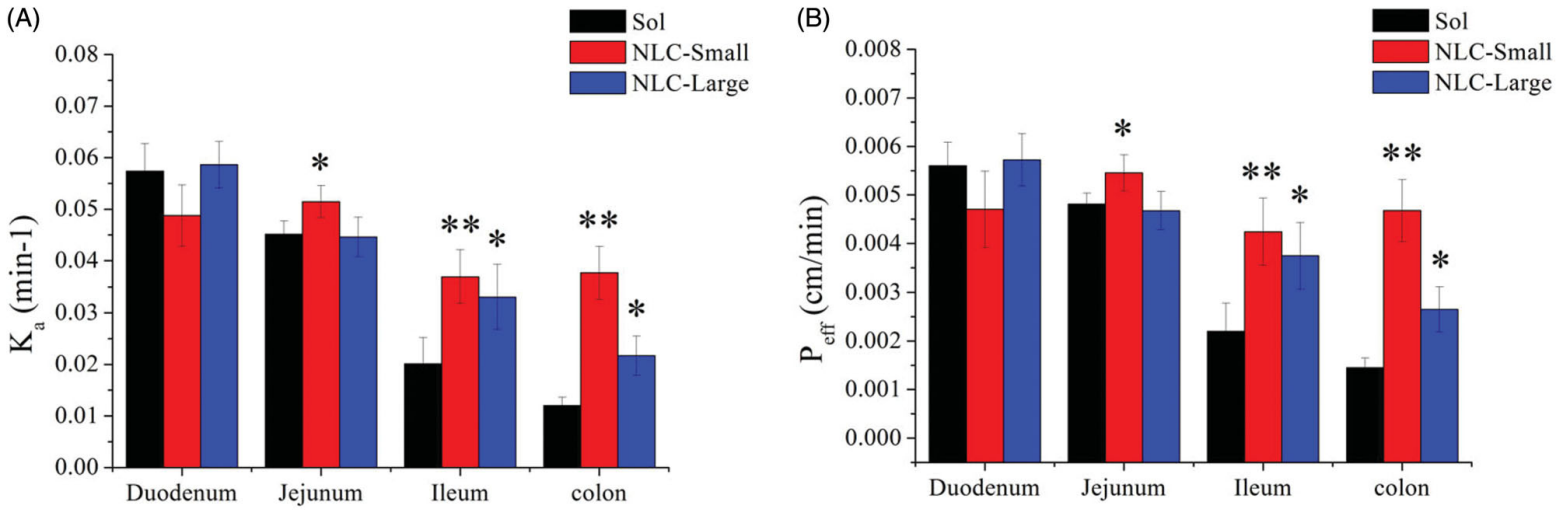
The *K*_a_ (A) and *P*_eff_ (B) obtained from *in situ* single-pass intestinal perfusion of REP-NLCs-Small, REP-NLCs-Large, and REP-Sol in duodenum, jejunum, ileum, and colon in rats (means ± SD, *n* = 3, *p<.05, **p<.01).

The absorption performances of REP-NLCs and REP-Sol in duodenum, jejunum, ileum, and colon were studied by an SPIP study. The obtained *K*_a_ and *P*_eff_ values are shown in Figure 3. Repaglinide could be absorbed well in the four intestinal segments from the result of repaglinide solution (REP-Sol), which was consistent with the feature of BCS II drug and determining results of *P*_eff_ values using SPIP method in rats for high-permeability compound (Zakeri-Milani et al., 2005). The *K*_a_ and *P*_eff_ values of duodenum and jejunum were significantly better than those of ileum and colon for REP-Sol, which was corresponding with Yin et al. (2012). The *K*_a_ and *P*_eff_ values of REP-NLC-Small size showed significant difference and improvement in the jejunum (*p* < .05), ileum (*p* < .01) and colon (*p* < .01) compared with REP-Sol. *K*_a_ and *P*_eff_ values of REP-NLC-Large size showed significant difference and improvement in the ileum (*p* < .05) and colon (*p* < .05) compared with REP-Sol. There were no significant differences for two kinds of REP-NLCs and REP-Sol in duodenum. It was interesting that there was high absorption performance for REP-NLC-Small size among duodenum, jejunum, ileum, and colon, and besides, compared with REP-NLC-Large size, REP-NLC-Small size showed significantly high absorption performances in jejunum (*p<* .05) and colon (*p* < .01).

The results showed that NLCs could significantly improve intestinal absorption of REP, and REP-NLC-Small size demonstrated better intestinal absorption potential than REP-NLC-Large size, which might be due to passing through epithelium easily for small particle size except for good permeability of mucus due to lower negative-charge surface as well as hydrophilicity of two formulations. Considering that the better intestinal absorption may cause higher drug concentration in plasma, we can understand the corresponding pharmacokinetic results.

REP-NLC-Small size demonstrated significantly better intestinal absorption ability than REP-NLC-Large size in jejunum and colon and two formulations was significantly better than REP-Sol in ileum and colon, which might be attributed to the features of differently intestinal segments. The gastrointestinal tract, consisting of esophagus, stomach, small intestine (duodenum, jejunum, and Ileum) and large intestine (colon and rectum) has approximately 6 m long with different diameters in different segments (Rabanel et al., 2012; Hua et al., 2015). The total surface area for intestine is about 200 m^2^. The surface is covered with columnar epithelial cells, including enterocytes, M cells contained in Peyer’s patch, and goblet cells secreting substance composing mucus, except for buccal and rectal with stratified squamous epithelium (Deli, 2009; Hwang & Byun, 2014). The major transport routes of drug absorption across enterocytes and interspersed M cells include passive paracellular transport by narrow channels and tight junctions, passive transcellular transport, transcellular transport mediated by uptake or efflux carrier, and transport via endocytosis (Balimane et al., 2000; Hwang & Byun, 2014). Tight junctions allow small and hydrophilic molecules to permeate through enterocytes (Balimane et al., 2000; Avdeef, 2001). NPs through tight junctions depend on opening junctions due to tightness (Rabanel et al., 2012). It is known that lipids possess absorption promoting features named as penetration enhancers by altering tight junction, such as medium chain fatty, bile salts, medium chain caprylic/capric, and long chain fatty acids (stearic acid and oleic acid) and so on, and MCT is more effective than long-chain triglycerides (LCTs) (Gaba et al., 2015; Niu et al., 2016). For endocytosis, the small carrier has good uptake (Pawar et al., 2014). All of the points above may result in better permeation performance and then improved bioavailability of REP-NLCs than REP-Sol.

**Figure 4.**
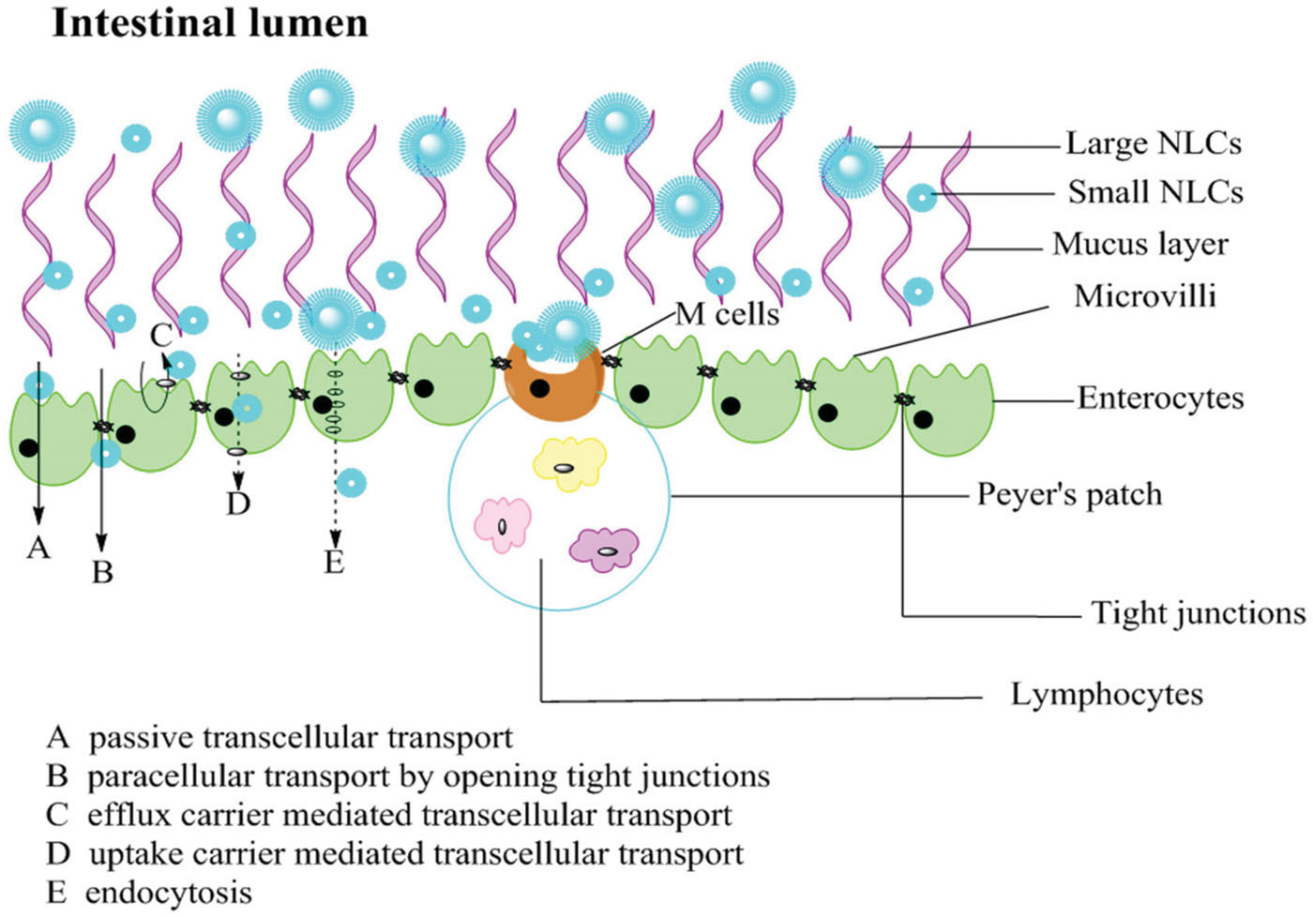
Schematic presentation of the mucus layer, intestinal epithelial cells layer and the transport routes of NLCs with different particles size.

Besides, considering the factor-particle size, the maximum uptake of enterocytes for particles size is 50–100 nm, while M cells can up to micrometer and ∼100 nm is more efficiently than larger size. Although M cell has only about 1% mucosal surface of intestine, its uptake ability predominates over enterocytes (Shakweh et al., 2004; Peng et al., 2010; Pawar et al., 2014). The amount of Peyer’s patch and M-cells increased sequentially in intestine is jejunum, ileum, and colon (Peng et al., 2010). These opinions may be the reasons for the *K*_a_ and *P*_eff_ value for REP-Sol, REP-NLC-Large, and REP-NLC-Small were significantly increased sequentially whether they were in jejunum or ileum or colon while had no significant difference in duodenum. Besides, the tightness of intercellular junction in jejunum and ileum is lower while in colon it is intermediate tightness and villous of small intestine increases surface area compared with colon without villous (Deli, 2009), which may explain that the absorption of NLC-Large size and REP-Sol were inferior in colon than in other intestinal segments while NLC-Small size in colon is relatively good for its small size and thus big contact area. The schematic diagram of absorption pathways of NLCs with different particles size in intestine tract is listed in Figure 4.

The result of better permeation in colon for NLC-Small size than NLC-Large size may give us an inspiration to prepare formulation with small particle size to cure colonic diseases to solve the problem of many drugs absorbed from upper gastrointestinal tracts.

## 4. Conclusions

In this study, repaglinide-loaded NLCs with different particles size of 79 nm and 325 nm were successfully prepared. The two formulations were same at zeta potential, shape, colloidal stability, and had no burst release phenomenon. SPIP study using gravimetric method developed successfully to correct drug concentration demonstrated the improved significantly membrane permeability for REP-NLC-Large in Ileum and colon and REP-NLC-Small in jejunum, ileum, and colon compared with REP-Sol. Besides, intestinal permeability of NLC-Large and NLC-Small also had significant differences in jejunum and colon, and NLC-Small was significantly better. The *in vivo* pharmacokinetics indicated that the bioavailability increased sequentially in REP-Sol, REP-NLC-Large, and REP-NLCs-Small, and the differences between each other were statistical significant. This study demonstrates the possibility to overcome mucus and epithelium barriers meantime for orally administered NPs and get possible candidate drugs formulation to cure colonic diseases by preparing low negative-charge NPs with 50–100 nm size.
